# Quantitative measurement of cartilage morphology in osteoarthritis: current knowledge and future directions

**DOI:** 10.1007/s00256-022-04228-w

**Published:** 2022-11-15

**Authors:** Wolfgang Wirth, Christoph Ladel, Susanne Maschek, Anna Wisser, Felix Eckstein, Frank Roemer

**Affiliations:** 1 Department of Imaging & Functional Musculoskeletal Research, Institute of Anatomy & Cell Biology, Paracelsus Medical University Salzburg & Nuremberg, Strubergasse 21, 5020 Salzburg, Austria; 2 Ludwig Boltzmann Inst. for Arthritis and Rehabilitation, Paracelsus Medical University Salzburg & Nuremberg, Salzburg, Austria; 3 Chondrometrics GmbH, Freilassing, Germany; 4 CHL4special Consultancy, Darmstadt, Germany; 5 Quantitative Imaging Center, Department of Radiology, Boston University School of Medicine, Boston, MA USA; 6 Department of Radiology, Universitätsklinikum Erlangen and Friedrich-Alexander-University Erlangen-Nürnberg (FAU), Erlangen, Germany

**Keywords:** Osteoarthritis, Cartilage loss, Knee, MRI

## Abstract

Quantitative measures of cartilage morphology (“cartilage morphometry”) extracted from high resolution 3D magnetic resonance imaging (MRI) sequences have been shown to be sensitive to osteoarthritis (OA)-related change and also to treatment interventions. Cartilage morphometry is therefore nowadays widely used as outcome measure for observational studies and randomized interventional clinical trials. The objective of this narrative review is to summarize the current status of cartilage morphometry in OA research, to provide insights into aspects relevant for the design of future studies and clinical trials, and to give an outlook on future developments. It covers the aspects related to the acquisition of MRIs suitable for cartilage morphometry, the analysis techniques needed for deriving quantitative measures from the MRIs, the quality assurance required for providing reliable cartilage measures, and the appropriate participant recruitment criteria for the enrichment of study cohorts with knees likely to show structural progression. Finally, it provides an overview over recent clinical trials that relied on cartilage morphometry as a structural outcome measure for evaluating the efficacy of disease-modifying OA drugs (DMOAD).

## Introduction

Osteoarthritis (OA) is a chronic disease and a leading cause of disability [1] that is characterized by alterations of all joint tissues, which eventually lead to joint failure and need for surgical joint replacement. The pathogenesis of OA involves all joint tissues, including cartilage, subchondral bone, and fibrocartilage such as menisci and labrum (depending on joint), joint capsule, ligaments, and surrounding muscles. Structural tissue damage results in illness perceived by the patient as pain, stiffness, and functional limitations [2, 3], with the knee being the most commonly affected joint [2, 4]. Current treatment guidelines focus on the management of symptoms [5–8], as no disease-modifying OA drug (DMOAD) has been approved by regulatory authorities yet. Since cartilage loss is considered one of the hallmark features of the disease, most DMOAD candidates aim to stop or slow cartilage loss or to stimulate cartilage growth [9].

Clinical trials evaluating DMOAD efficacy traditionally relied on quantitatively measured radiographic joint space width (JSW), considered a surrogate for cartilage loss and integrity. Radiography is, however, not able to directly depict the articular cartilage and radiographically measured JSW represents a composite measure as the joint space is maintained by both cartilage and meniscus [10, 11]. Magnetic resonance imaging (MRI), in contrast, provides a direct depiction of all tissues, including the articular cartilage, and is now widely used in OA research and clinical trials for pharmaceutical development [12].

Since Peterfy et al. reported that an accurate and reproducible volumetric quantification of articular cartilage in the knee is possible from 3D MRI in 1994 [13], numerous observational and interventional studies employed quantitative measurements of cartilage morphology [14, 15]. Developments in this field have been driven by the need for reliable and sensitive MRI-based imaging biomarkers suitable for replacing JSW as structural efficacy outcome measure in clinical trials. Important insights on feasibility, reliability, and validity of MRI have been made possible through its application in large longitudinal studies (e.g. progressive vs. non-progressive disease). One example is the Osteoarthritis Initiative (OAI), which provides a large variety of data for a total of 4796 participants, including high-resolution knee MRIs suitable for quantitative cartilage analyses [15].

The objective of this narrative review is to summarize the current status of quantitative cartilage morphology measurements (i.e., cartilage morphometry) in OA research, to provide insights into aspects relevant for the design of future studies and clinical trials, and to give an outlook on future developments. Since cartilage morphometry is mainly used in the context of knee OA, this review focuses on the knee. The methodologies used for cartilage morphometry can, however, also be translated to other joints.

## Image acquisition

Cartilage morphometry requires high resolution, 3D gradient echo sequences that provide a sufficient contrast between the articular cartilage, the subchondral bone, the menisci, and intraarticular fluid. Earlier studies have validated MRI sequences such as T1-weighted spoiled gradient echo (SPGR, GE) MRI, fast low angle shot (FLASH, Siemens), fast field echo (FFE, Philips), or water selective cartilage (WATSc, Philips) MRI against external standards (e.g., water displacement or CT arthrography) and reported a high accuracy for 3D cartilage volume, thickness, and area measurements (Table 1) [13, 16–22]. In order to reduce chemical shift artefacts, all of these MRI sequences use selective water excitation as a means of fat suppression [13, 16–22]. These MRI sequences have been shown to allow reproducible assessment of cartilage morphology parameters and are therefore widely used in observational and interventional studies [14, 23, 24]. More recently, MRI sequences such as 3D double echo at steady state (DESS, Siemens) with water excitation (or comparable protocols from other vendors) became available [25–27], which additionally allow to estimate the cartilage T2 relaxation time as a measure of cartilage composition and to perform semi-quantitative assessments of some joint pathologies [28]. The magnetic field strength should be at least 1.5 T in order to ensure a sufficient signal-to-noise ratio and resolution at a reasonable scan time; precision errors have been reported to be smaller for 3 T MRI than for 1.5 T MRI [29]. In addition, scan times (i.e., burden on the patient) are reduced at 3 T MRI.

**Table 1:** Overview over technical MRI validation studies

Authors	Year	Joint	Scanner	Resolution	Sample size	External reference	Results
Marshall et al.^1^	1995	Phantom	General Electrics 1.5 T	In-plane: 0.29 × 0.78 mm or 0.29 × 0.59 mm; slice thickness: 1.5 mm	4 patella phantoms × 5 observer × 5 measurements	Water displacement	Mean absolute difference (cartilage volume): 1.7 + / − 1.3% for 1.0 mm slices, 3.5 + / − 2.5% 1.5 mm slices, 6.4 + / − 2.5% for 2.0 mm slices, 22.7 + / − 1.9% for 3.0 mm slices
		Bovine joints	General Electrics 1.5 T	0.29 × 0.78 × 1.5 mm	4 joints × 5 observer	Caliper	Correlation (cartilage thickness): *r* = 0.80 Mean systematic difference (cartilage thickness): − 3.2 to 5.8%
Eckstein et al.^2^	1996	Human joint specimens	Siemens 1.5 T	0.31 × 0.31 × 2 mm	8	Anatomical sections	Mean systematic difference (cartilage volume): patella: − 3.6%, femur: + 3.8% medial tibia: − 4.2%, lateral tibia: + 1.1%
Kladny et al.^3^	1996	Human joint specimens	Siemens 1.5 T	1.5 mm slice thickness	14	Histological sections	Correlation (cartilage thickness): *r* = 0.96 Mean systematic difference (cartilage thickness): tibia: 0.12 + / − 0.28 mm; 8.4%
Dupuy et al.^4^	1996	Human joint specimens/knee joint replacement	General Electrics 1.5 T	0.62 × 0.83 × 0.3 mm	6	Water displacement	Accuracy (cartilage volume): 93% Correlation (cartilage volume): *r* = 0.99
Schnier et al.^5^	1997	Human joint specimens	Siemens 1.5 T	0.31 × 0.31 × 2 mm	10	CT arthrography	Mean systematic difference (cartilage volume): patella: 2.5%, femur: + 2.3%, medial tibia: 0.7%, lateral tibia: 0.3%
Cohen et al.^6^	1999	Human joint specimens	General Electrics 1.5 T	0.47 × 0.47 × 1.0 mm	6	Stereo-photo-grammetry	Systematic difference (cartilage thickness, from registration of subchondral bone areas): patella: 0.12 + / − 0.22 mm, femur: 0.17 + / − 0.15 mm, tibia: 0.04 + / − 0.33 mm
Bauer et al.^7^	2006	Porcine joints	General Electrics 1.5 T/3 T	0.19 × 0.39 × 1.5 mm	18	Water displacement	Mean systematic difference/correlation (cartilage volume): 1.5 T FS patella: − 0.18 mL, *r* = 0.88 1.5 T FS femur: − 1.05 mL, *r* = 0.88 3 T FS patella: − 0.03 mL, *r* = 0.90 3 T FS femur: − 0.05 mL, *r* = 0.99 3 T WE patella: − 0.03 mL, *r* = 0.95 3 T WE femur: − 0.22 mL, *r* = 0.95
Bowers et al.^8^	2008	Human joint specimens	Siemens 3 T	0.31 × 0.31 × 1.5 mm	1 specimens × 7 scans	Laser scan	Mean absolute error (cartilage thickness): manual segmentation: total: 4.07% Femur: 5.4% Tibia: 0.2% Live-wire segmentation: Total: 7.46% Femur: 8.9% Tibia: 3.1%
Xing et al.^9^	2011	Porcine joints	Siemens 3 T	0.4 × 0.4 × 1.0 mm	20 knees × 2 protocols × 2 scans	Water displacement	Correlation (cartilage volume): *r* = 0.90–0.98 Systematic pair-wise difference: FLASH MRI: Patella: 1.2% Trochlea: 0.4% Medial femur: − 0.6% Lateral femur: − 0.5% MEDIC MRI: Patella: − 1.1% Trochlea: 0.6% Medial femur: 2.5% Lateral femur: 2.8%
Peterfy et al.^10^	1994	Knee replacement surgery	General Electrics 1.5 T	Field of view: 14–16 cm Slice thickness: 1.5–3–0 Matrix 256 × 128–256	8	Water displacement	Correlation (cartilage volume): *r* = 0.99 Mean systematic difference: GRASS MRI: 0.40 mL + / − 1.1 mL (8.2%) Spoiled GRASS MRI: 0.31 mL + / − 0.08 mL (5.9%)
Graichen et al.^11^	2004	Knee replacement surgery	Siemens 1.5 T	0.31 × 0.31 × 1.5 mm	21	Water displacement/aluminium foil for areas	Mean absolute difference/mean systematic difference/correlation: Subchondral bone area: Patella: 7.7%/ − 4.7%/0.94 Medial Tibia: 8.5%/ − 7.6%/0.94 lateral tibia: 7.3%/ − 7.0%/0.97 Cartilage volume: Patella:6.6%/5.1%/0.99 Medial Tibia: 11.5%/ − 3.1%/0.98 lateral tibia: 10.5%/3.6%/0.95 Cartilage thickness: Patella:4.3%/ − 1.8%/0.97 Medial Tibia:12.3%/0.4%/0.72 lateral tibia: 10.0%/ − 4.7%/0.94
Cromer et al.^12^	2013	Porcine joints	General Electrics 3 T	0.5 × 0.5 × 2.2 mm	8	Water displacement	Correlation (cartilage volume): *r* = 0.75 Mean systematic difference (total joint volume): 0.33 + / − 0.18 mL
Jaremko et al.^13^	2007	Porcine joints	Siemens 1.5 T	0.3 × 0.3 × 1.2 mm	15	Water displacement	Mean systematic difference (cartilage volume of patella fragments): − 0.007 + / − 0.056 mL or –1.6% + / − 13.2%
Burgkart et al.^14^	2001	Knee replacement surgery	Siemens 1.5 T	0.31 × 0.31 × 1.2 mm	8	Water displacement	Correlation of longitudinal change (cartilage volume): *r* = 0.98 Mean systematic difference of longitudinal change (cartilage volume): − 7 to − 27%
Eckstein et al.^15^	1998	Human joint specimens	Siemens 1.5 T	0.31 × 0.31 × 2 mm	8	CT arthrography	Correlation/mean systematic difference (cartilage volume): Patella: *r* = 1.0/ + 2.5% Femur: *r* = 0.95/ + 3.5% Medial tibia: *r* = 0.94/ + 1.7% lateral tibia: *r* = 0.98/ − 0.8%
Haubner et al.^16^	1997	Human joint specimens	Siemens 1.5 T	0.31 × 0.31 × 2 mm	6	CT arthrography	Mean systematic difference (cartilage volume): Patella: 3.5% Femur: 5.9% Medial tibia: 5.7% Lateral tibia: 9.8%

^1^Marshall KW, Mikulis DJ, Guthrie BM. Quantitation of articular cartilage using magnetic resonance imaging and three-dimensional reconstruction. *J Orthop Res*. 1995;13(6):814–823

^2^Eckstein F, Gavazzeni A, Sittek H, Haubner M, Losch A, Milz S, et al. Determination of knee joint cartilage thickness using three-dimensional magnetic resonance chondro-crassometry (3D MR-CCM). *Magn Reson*. 1996;36(0740–3194):256–265

^3^Kladny B, Bail H, Swoboda B, Schiwy-Bochat H, Beyer WF, Weseloh G. Cartilage thickness measurement in magnetic resonance imaging. *OsteoarthritisCartilage*. 1996;4(1063–4584):181–186

^4^Dupuy DE, Spillane RM, Rosol MS, Rosenthal DI, Palmer WE, Burke DW, et al. Quantification of articular cartilage in the knee with three-dimensional MR imaging. *AcadRadiol*. 1996;3(1076–6332):919–924

^5^Schnier M, Eckstein F, Priebsch J, Haubner M, Sittek H, Becker C, et al. [Three-dimensional thickness and volume measurements of the knee joint cartilage using MRI: validation in an anatomical specimen by CT arthrography]. *Rofo*. 1997;167(5):521–526

^6^Cohen ZA, McCarthy DM, Kwak SD, Legrand P, Fogarasi F, Ciaccio EJ, et al. Knee cartilage topography, thickness, and contact areas from MRI: In- vitro calibration and in-vivo measurements. *Osteoarthr Cartil*. 1999;7(1063–4584):95–109

^7^Bauer JS, Krause SJ, Ross CJ, Carballido-gamio J, Ozhinsky E, Link TM. Volumetric cartilage measurements of porcine knee at 1.5-T and 3.0-T MR imaging: evaluation of precision and accuracy. 2006;241(2):399–406

^8^Bowers ME, Trinh N, Tung GA, Crisco JJ, Kimia BB, Fleming BC. Quantitative MR imaging using &quot;LiveWire&quot; to measure tibiofemoral articular cartilage thickness. *Osteoarthr Cartil*. 2008;16(10):1167–1173

^9^Xing W, Sheng J, Chen W, Tian J, Zhang L, Wang D. Reproducibility and accuracy of quantitative assessment of articular cartilage volume measurements with 3.0 Tesla magnetic resonance imaging. *Chin Med J (Engl)*. 2011;124(8):1251–1256

^10^Peterfy CG, van Dijke CF, Janzen DL, Gluer CC, Namba R, Majumdar S, et al. Quantification of articular cartilage in the knee with pulsed saturation transfer subtraction and fat-suppressed MR imaging: optimization and validation. *Radiology*. 1994;192(0033–8419):485–491

^11^Graichen H, Eisenhart-Rothe R V., Vogl T, Englmeier KH, Eckstein F. Quantitative assessment of cartilage status in osteoarthritis by quantitative magnetic resonance imaging: technical validation for use in analysis of cartilage volume and further morphologic parameters. *Arthritis Rheum*. 2004;50(0004–3591):811–816

^12^Cromer MS, Foster SL, Bourne RM, Fransen M, Fulton R, Wang SC. Use of 3 T MRI and an unspoiled 3D fast gradient echo sequence for porcine knee cartilage volumetry: Preliminary findings. *J Magn Reson Imaging*. 2013;38:245–250

^13^Jaremko JL, Maciejewski CM, Cheng RWT, Ronsky JL, Thompson RB, Lambert RGW, et al. Accuracy and reliability of MRI vs. laboratory measurements in an ex vivo porcine model of arthritic cartilage loss. *J Magn Reson Imaging*. 2007;26:992–1000

^14^Burgkart R, Glaser C, Hyhlik-Dürr A, Englmeier KH, Reiser M, Eckstein F, et al. Magnetic resonance imaging-based assessment of cartilage loss in severe osteoarthritis: accuracy, precision, and diagnostic value. *Arthritis Rheum*. 2001;44(0004–3591):2072–2077

^15^Eckstein F, Schnier M, Haubner M, Priebsch J, Glaser C, Englmeier KH, et al. Accuracy of cartilage volume and thickness measurements with magnetic resonance imaging. *Clin Orthop Relat Res*. 1998;(352):137–148

^16^Haubner M, Eckstein F, Schnier M, Losch A, Sittek H, Becker C, et al. A non-invasive technique for 3-dimensional assessment of articular cartilage thickness based on MRI. Part 2: Validation using CT arthrography. *Magn Reson*. 1997;15(0730-725X):805–813

Most current cartilage morphometry studies employ an in-plane resolution of ~ 0.3 mm and a slice thickness between 1.0 and 1.5 mm, which lies within the range suggested in the OARSI clinical trial recommendations [23]. This resolution has been shown to provide a satisfactory accuracy and reproducibility and a high sensitivity to change [14]. Thinner slices have been associated with smaller precision errors [29] but were not observed to translate into a relevant improvement of the sensitivity to change in a study comparing the sensitivity to change between sagittal DESS MRIs with 0.7 mm vs. 1.4 mm slice thickness [30].

**Fig. 1:**
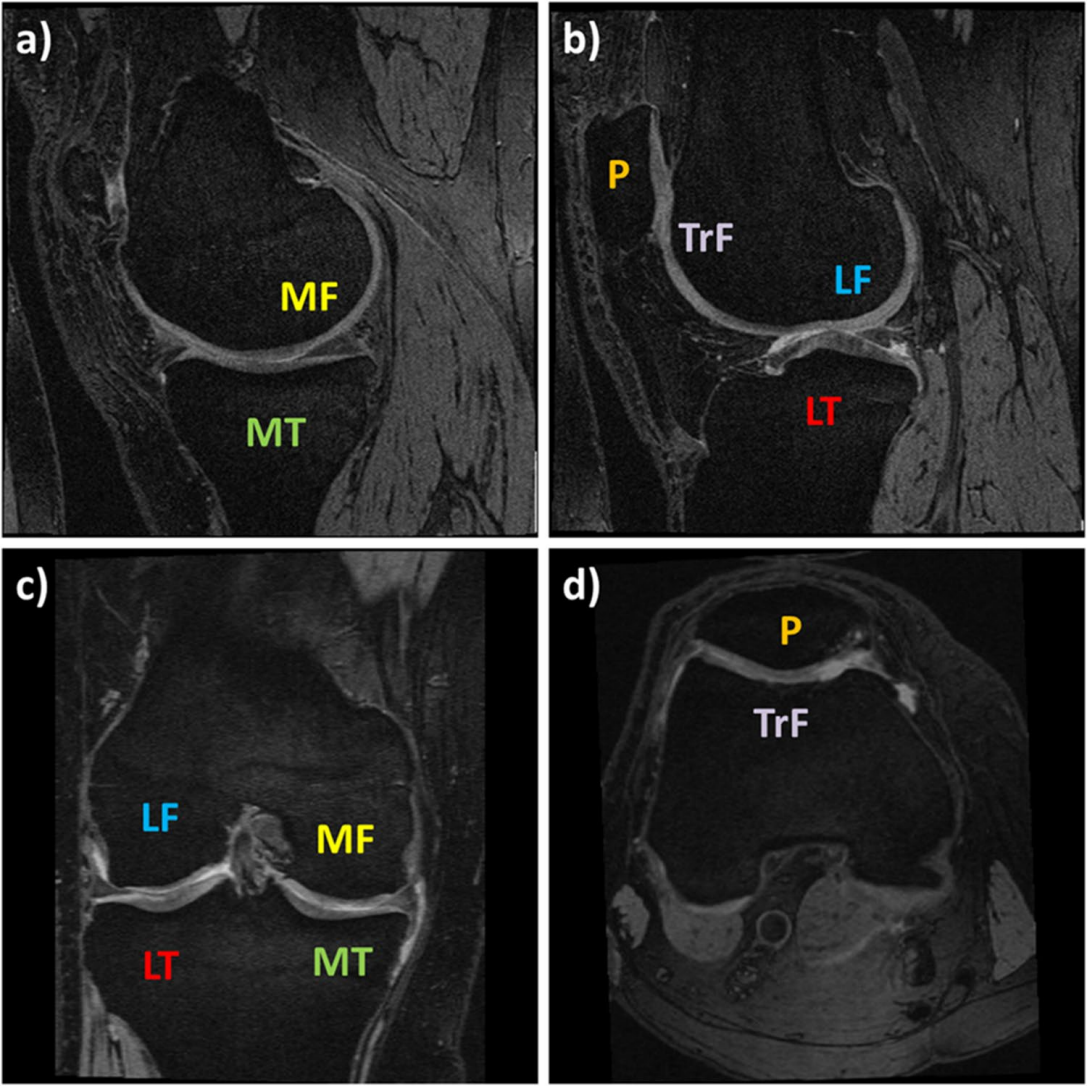
Illustration showing the MRI orientations and the cartilages that can be analyzed from these orientations. **a** Sagittal MRI slice through the medial compartment, **b** sagittal MRI slice through the lateral compartment, **c** coronal MRI slice, **d** axial MRI slice (P: patella, TrF: trochlea of the femur, MF: medial femur, LF: lateral femur, MT: medial tibia, LT: lateral tibia). The double echo at steady state (DESS) MRIs were provided by the Osteoarthritis Initiative image data base

Because partial volume effects impair the precision and accuracy of quantitative cartilage morphology measurements, the slice orientation needs to be chosen perpendicular to the structures of interest. The sagittal orientation is the most versatile one, as it can be used for analyzing the cartilages in the whole tibiofemoral as well as the patellofemoral joint (Fig. 1). Coronal orientations provide a good depiction of the central, weight-bearing part of the tibiofemoral joint cartilages, but are not suitable for the patellofemoral joint or the posterior aspects of the femoral condyles (Fig. 1). These cartilages can, instead, be well analyzed from axial scans (Fig. 1). Isotropic or near-isotropic, high-resolution scans provide the advantage of having only small partial volume effects for all imaging planes. They require longer scan times or provide only a limited signal-to-noise ratio, but allow reducing the impact of partial-volume effects on cartilage morphometry when imaging spherical joints such as the hip. 3D fast spin-echo MRI protocols such as CAIPIRINHA (Controlled Aliasing in Parallel Imaging Results in Higher Acceleration) provide isotropic scans with reasonable signal-to-noise ratio at reasonable acquisition times due to higher acceleration factors [31–34]. The resolution of such scans is, however, typically not as high (≥ 0.5 mm) than that used for cartilage morphometry from gradient echo MRI (~ 0.3 mm) and the accuracy and precision of cartilage morphometry based on such this or comparable fast spin-echo sequences still needs to be evaluated (e.g., against external standards or previously validated sequences).

In longitudinal studies, the scan parameters (e.g., MRI system, coils) need to remain consistent for all time points of the same subject, because a change of equipment during a study typically results in a systematic offset that may exceed the longitudinal change [27, 29, 35]. Multi-center studies relying on equipment from different vendors at different sites have shown consistent cartilage thickness values and precision errors [27, 36], e.g. by the application of phantoms and cross-validation of the acquired images [37]. Such trials have used different, but comparable 3D sequences that allow for cartilage quantification such as FLASH/VIBE/SPGR/T1-FFE/WATSc protocols and DESS-like protocols, which have been shown to provide a comparable sensitivity to change [30]. Current clinical trials recruit patients at multiple centers using MRI protocols that are tailored to the equipment available at the respective site [31]. Before the scanning of subjects starts, all centers need to be qualified and the image quality approved to ensure a sufficient quality of the scans.

Cartilage morphometry depends heavily on the standardization and quality of the MRIs. Studies using cartilage morphometry as an outcome measure should therefore prepare detailed, standardized imaging protocols addressing all imaging-related aspects. The OAI Imaging protocol can serve as reference when developing the imaging protocol for current studies (see https://nda.nih.gov/static/docs/StudyDesignProtocolAndAppendices.pdf) [26]. In addition, for randomized clinical DMOAD trials, an imaging charter is required (https://www.fda.gov/regulatory-information/search-fda-guidance-documents/clinical-trial-imaging-endpoint-process-standards-guidance-industry) [39]. As repetitions of unsuitable/inappropriate scans may be difficult or even impossible (e.g., pre-treatment baseline scans cannot be repeated after the start of the treatment), a timely quality check of the scans is of great importance for clinical trials. Aspects that should be covered during the quality assurance process include:

- Complete coverage of the whole joint
- Adequate orientation of the scans
- Absence of artefacts (e.g., motion, metal) in the region(s) of interest
- Overall image quality (e.g., image contrast, signal-to-noise ratio)
- Imaging parameters consistent with the imaging protocol
- Correct joint imaged across visits

## Image analysis

**Fig. 2:**
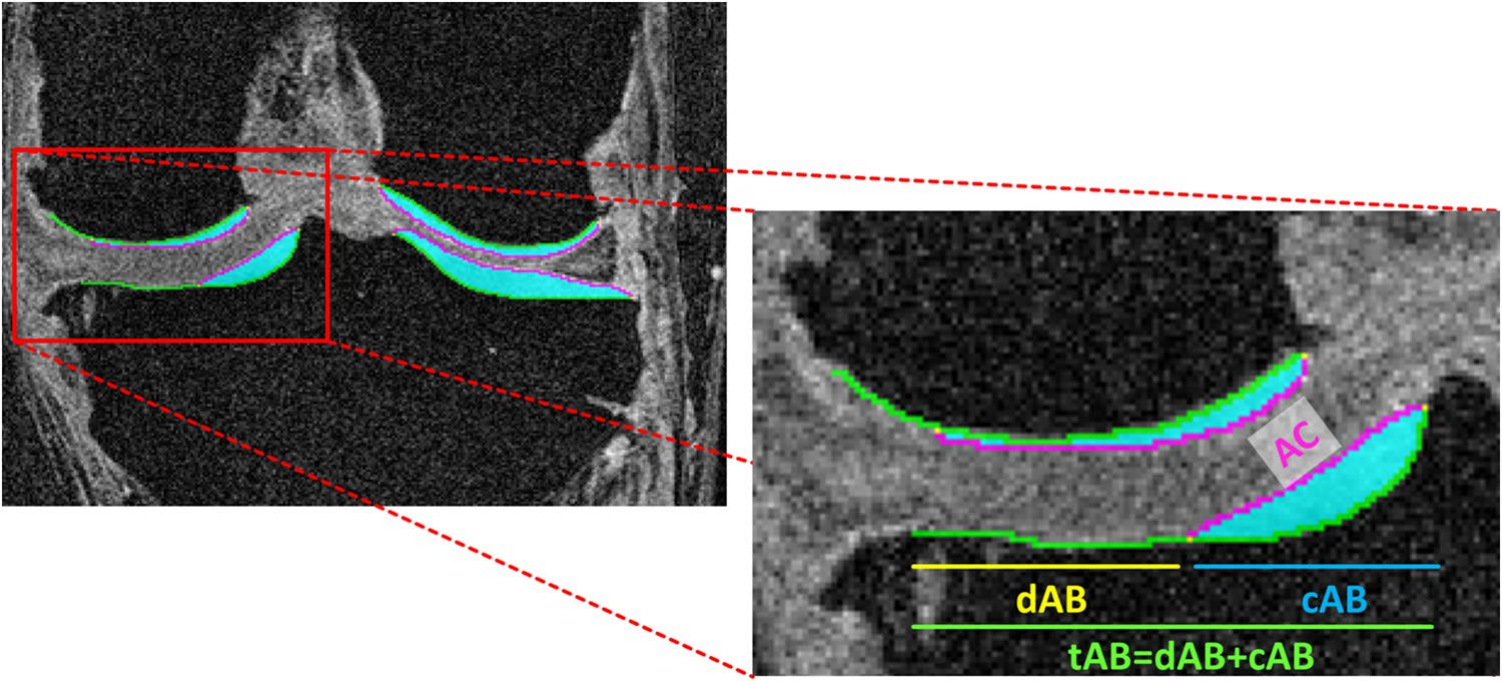
Illustration showing the segmentation of cartilages areas in a coronal FLASH MRI from the Osteoarthritis Initiative (tAB = total area of subchondral bone, AC = cartilage surface area, dAB = denuded area of subchondral bone, cAB = covered area of subchondral bone)

Cartilage morphometry requires segmentation of the articular cartilage as a basis for calculating quantitative measures (e.g., thickness, volume, or area, Table 2, Fig. 2). The manual segmentation of cartilage by expert readers is still considered the gold standard [40]. It has been employed as an outcome measure in observational studies and clinical trials [14, 15, 38, 41] and has also been used for training automated segmentation techniques as well as a reference for evaluating the performance of automated methods [42–47]. Numerous semi- and fully automated segmentation methodologies have been proposed over the last 2 decades for automatically segmenting the articular cartilages, ranging from b-spline snakes [48], edge-tracking [49], or local area cartilage segmentation [50] over shape-based methods [45] and clustering approaches [51] to deep learning-based techniques [46]. Ebrahimkani et al. recently published a review on segmentation techniques used in OA research [47]. All segmentation techniques, automated and manual, should be thoroughly validated to ensure the reliability and accuracy of the results. A centralized quality control of all segmentations is recommendable for manual cartilage segmentations and has recently been suggested to also improve the sensitivity to change when applied to deep learning-based cartilage segmentations [52].

**Table 2:** Morphological cartilage measures

Category	Measure	Abbreviation	Unit
Cartilage area	Total area of subchondral bone: premorbid subchondral bone area, excluding peripheral osteophytes	tAB	cm^2^
	Covered area of subchondral bone: part of the tAB that is covered with cartilage	cAB	cm^2^ or in % der tAB
	Denuded area of subchondral bone: part of the tAB that is not covered with cartilage (full thickness cartilage loss)	dAB	cm^2^ or in % der tAB
	Area of cartilage surface	AC	cm^2^
Cartilage volume	Cartilage volume	VC	µL, mL, or mm^3^
Cartilage thickness	Cartilage thickness over tAB: denuded areas are counted as having 0 mm cartilage thickness	ThCtAB	mm
	Cartilage thickness over cAB: denuded areas are excluded	ThCcAB	mm

In addition to high quality segmentation, appropriate and validated software is required for computing quantitative 3D cartilage measures. Simplistic measures (e.g., cartilage volume) can be derived from cartilage segmentations using off-the-shelf software (e.g., ImageJ), whereas the computation of more specialized measures (e.g., cartilage thickness over the total area of subchondral bone) typically requires dedicated and validated software solutions. An overview over the variety of measures (e.g., cartilage thickness, volume, total area of subchondral bone, cartilage surface area) that can be derived from cartilage segmentation has been published [53] (Table 2, Fig. 2). Several recent clinical trials used cartilage thickness measures as primary or secondary outcomes [38, 41, 54, 55], while others still rely on cartilage volume as an outcome measure [56, 57].

**Fig. 3:**
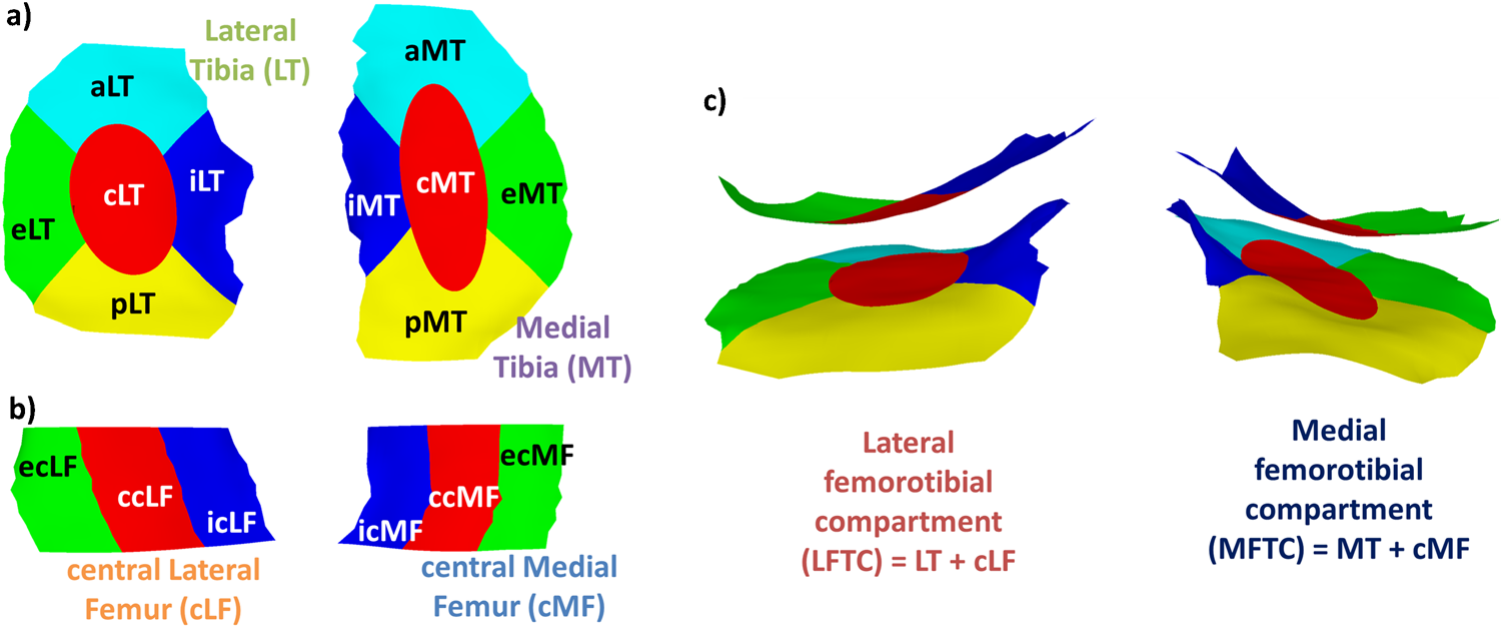
3D reconstruction of the total area of subchondral bone showing the femorotibial cartilage regions and the 16 femorotibial cartilage subregions (prefix a/c/e/i/p: anterior/central/external/internal/posterior subregion of the respective cartilage plate, e.g., cMT: central subregion of MT). a—Shows the tibial cartilage subregions, b—shows the weight-bearing, central femoral cartilage subregions, c—shows the medial and the lateral femorotibial compartment

Cartilage morphometry can be derived from individual cartilage plates but also from aggregate regions (e.g., medial femorotibial compartment), defined regions of interest (e.g., weight-bearing, central part of the medial femoral condyle), or for cartilage subregions (Fig. 3) [53, 58]. Larger regions of interest (e.g., medial femorotibial compartment) have been reported to show a smaller variability of the longitudinal change in morphometric measures than smaller regions of interest (e.g., central subregion of the medial tibia) [59]. Smaller regions of interest may, instead, be advantageous in case of risk factors for progression, that are expected to specifically affect defined regions of interest (e.g., meniscus extrusion [60] or tears [61]). The decision on a specific region of interest selected for measuring the outcome should generally be aligned with the enrollment criteria, in order to avoid too broad or too narrow regions of interest (e.g., medial instead of total femorotibial joint cartilage thickness as outcome measure when selecting knees with medial joint space narrowing only).

Location-independent measures of change in cartilage morphology such as ordered values of subregional change [62–64] or thinning and thickening scores [65, 66] have been developed to remove the link between the magnitude of change and the location of change and to thereby focus on the regions with the greatest magnitude of thickness loss or gain, wherever it is located within the joint. Ordered values of subregional change can be computed by ordering the change observed in each of the 16 subregions within each knee in ascending order, whereas thinning or thickening scores can be computed by summing all negative or positive changes within each knee [66]. Location-independent measures have been shown to be highly sensitive to differences of change between groups and also to be sensitive to treatment effects induced by DMOAD candidates [64, 67, 68]. Moreover, they allow separate assessments of the efficacy of DMOAD candidates for reducing cartilage thickness loss (thinning score) and for stimulating cartilage growth (thickening score).

## Sensitivity of cartilage morphometry

Cartilage morphometry has been shown to be sensitive to OA-related change in longitudinal studies [14]. The sensitivity to change has been reported to depend on the participant selection criteria, the length of the follow-up period, and on the outcome measure [14, 69–72] and has also been suggested to be greater for cartilage morphometry than for radiographic JSW measurements [14, 73, 74]. The standardized response mean (SRM, i.e., the ratio between the magnitude of change and the standard deviation of change) is frequently used as a measure of the sensitivity to change in knee OA studies. Furthermore, cartilage morphometry has been shown to be sensitive to differences in change between groups, e.g., in observational studies evaluating the impact of risk factors for progression [71, 75–80]. More recently, quantitative cartilage morphology measures have been reported to be sensitive to treatment-related effects, by demonstrating a dose–response effect of femorotibial cartilage thickness change in the Sprifermin phase 2 trial over 2 and 5 years [41, 81, 82].

## Predictors of structural progression

Cartilage morphometry is widely used in longitudinal studies to evaluate the impact of risk factors on cartilage loss [75–78, 80], to characterize the magnitude and the pattern of cartilage loss in different cohorts [15, 76, 83, 84], and to study the efficacy of treatment interventions on slowing or stopping cartilage loss or on stimulating cartilage growth [38, 41, 54–56]. Because early stages of OA are not characterized by substantial cartilage loss but by alterations of the cartilage composition and matrix integrity [85], cartilage morphometry is mainly used in cohorts with definite signs of radiographic OA (e.g., osteophytes, joint space narrowing).

Kellgren and Lawrence grades are typically used to distinguish between knees with (KLG 2–4) vs. without (KLG 0–1) definite radiographic OA [86]. But even in knees with definite radiographic OA (KLG 2–4), cartilage tissue loss is a slow process [79], in particular in knees with KLG 2, in which not only thinning (i.e. cartilage degradation) but also thickening of cartilage (i.e. cartilage swelling and/or potential anabolic tissue response) has been observed to take place simultaneously [87]. Numerous studies and projects therefore investigated factors predictive of subsequent progression that would allow enriching clinical trials with knees likely to exhibit accelerated structural progression and that are therefore in need of a structure-modifying treatment. The FNIH-funded “Osteoarthritis Biomarkers” (https://fnih.org/our-programs/biomarkers-consortium/osteoarthritis-project) and “PROGRESS OA” (https://fnih.org/our-programs/biomarkers-consortium/programs/progress-oa) projects aimed for the identification and the regulatory approval of prognostic biomarkers for structural and/or symptomatic OA disease progression [88]. IMI-APPROACH (https://www.approachproject.eu/), a 2-year, European, prospective follow-up cohort project, collected conventional and novel clinical, imaging, and biochemical (bio)markers to validate and refine predictive machine-learning models for pre-identified and new progressor phenotypes based on these markers [89].

Radiography is traditionally used for the selection of participants for OA studies and KL grades have been shown to perform well as predictors of cartilage thickness loss [79]. Cartilage thickness changes in the medial/lateral femorotibial compartment have been reported to amount to + 0.1%/ + 0.2% in KLG 0 knees without risk factors for developing OA (healthy reference group), to − 0.3%/ − 0.2% in KLG 2 knees, to − 1.2%/ − 0.8% in KLG 3 knees, and to − 2.0%/ − 1.5% in KLG 4 knees over one year [79]. Knees with KLG 3 or 4 were reported to show more cartilage thickness loss than KLG 0 knees in this study [79], but these changes were still below the magnitude of intra- and inter-reader precision errors determined in a meta-analysis (coefficient of variation for both: 3%) [90]. This may be explained by the fact that KL grades are composite scores that take several OA-related radiographic features into account and do not allow a selection based on the predominantly affected compartment. Furthermore, recent studies reported that knees with KLG 2, which are typically included in treatment trials, not necessarily have cartilage damage [91]. This may be the case in up to one third of the KL2 knees included [92]. Inclusion of patients without cartilage damage in DMOAD trials assessing efficacy of a cartilage-anabolic compound is, however, not recommended, because there is neither an actual need for cartilage treatment nor are they likely to develop cartilage loss over the course of the trial.

Radiographic JSN scores [93] have been reported to be a strong predictor of cartilage loss in the narrowed compartment [94]. They have recently been successfully used in combination with KL grades to enrich the population of the ROCCELLA trial with knees likely to show medial compartment progression [38, 95], but are not only affected by cartilage status but also by meniscus integrity and extrusion [10].

MRI-based ordinal expert scoring such as ROAMES and MOAKS not only allows to identify knees that should not be included in clinical trials (e.g., because of subchondral insufficiency fracture) [96], but also to specifically select knees likely to show subsequent cartilage thickness loss (e.g., based on size and depth of cartilage damage or presence of inflammation) [97]. Besides selecting knees with a high likelihood of showing cartilage loss, it is crucial for clinical trials to consider the mode-of-action of the respective compound, in order to ensure that the DMOAD candidate is effective in the knees included in the cohort. MRI-based scorings may allow to select knees likely to be susceptible for treatment effects of a DMOAD candidate more specifically than radiographic measures (e.g. based on MRI-based extent of cartilage damage instead of radiographic JSN), but also to exclude knees, in which a treatment effect seems unlikely because of strong biomechanical factors (e.g., meniscus root tears) or because of too severe cartilage damage.

A detailed overview reporting the magnitude of change and the sensitivity to change in different cohorts with various selection criteria and observation period lengths has been published [14]. Short-term studies (e.g., 3 or 6 months) have been reported to be at high risk of failure because of insufficient longitudinal change [70]. Most studies therefore choose observation periods of 12 months or longer [14].

## Overview over recent clinical trials

Several recent clinical trials included cartilage morphometry as an outcome measure. The FORWARD study, a phase 2 trial evaluating the efficacy and safety of Sprifermin as DMOAD in 549 patients with tibiofemoral OA, relied on manual, quality-controlled cartilage segmentations from coronal gradient echo MRI that were acquired using 1.5 T or 3 T MRI scanners from different manufacturers (slice thickness: 1.5 mm, in-plane resolution: ~ 0.3 mm) for assessing the structural efficacy [41, 82]. This trial reported statistically significant, dose-dependent increases in the total femorotibial cartilage thickness over 2 and 5 years when compared against placebo (difference vs. placebo in highest dose group: 0.05 mm, 95% CI: [0.03, 0.07]mm) [41, 82]. Statistically significant dose–response effects were also observed for medial and lateral femorotibial cartilage thickness using cartilage morphometry, while radiographic JSW showed such effects only in the lateral but not in the medial compartment. A post hoc analysis reported a dose–response effect from automated cartilage segmentations that was consistent with the dose–response effect observed in the primary analysis [81]. Despite the structural efficacy, the FORWARD trial was not able to demonstrate clinical translation of Sprifermin in the full cohort (i.e., pain reduction). A recent post hoc analysis of a subset of FORWARD participants with severe OA (severe pain and low JSW) at baseline suggested, however, that Sprifermin may not only have structure-modifying but also symptom modifying efficacy, indicating that future studies evaluating Sprifermin should potentially focus on more advanced stages of OA [98].

The TPX-100–5 study used sagittal gradient echo MRIs acquired on 1.5 T MRI scanners from different manufacturers (slice thickness: 1.5 mm, in-plane resolution: ~ 0.3 mm) for evaluating the structural efficacy of intraarticular TPX-100 as potential DMOAD in knees with patellofemoral cartilage defects. The TPX-100–5 study reported clinically meaningful functional benefits over 1 year, while no effects on cartilage thickness from manual, quality-controlled segmentations could be observed [99]. This was explained by the limited magnitude of cartilage loss observed in the 93 subjects. A post hoc analysis performed in 78 of the 93 knees reported, however, a weak correlation between cartilage thickness change and changes in bone shape in the medial femoral condyle of knees treated with TPX-100 [55].

The efficacy and safety of MIV-711 as a potential DMOAD was evaluated over 26 weeks in 244 participants with primary knee OA [54]. When compared to placebo, the study observed no efficacy with regard to pain, but reported a significant reduction of central femoral cartilage thickness loss for the lower-dose group (100 mg MIV-711) as well as a treatment effect on bone shape for both treatment groups (100 mg & 200 mg MIV-711). The change in the central femoral cartilage thickness observed in the MIV-711, 200 mg group as well as the change observed in the central medial tibial cartilage thickness did, however, not differ from that observed for the placebo group. This study utilized statistical shape modelling methods for assessing cartilage loss, which were applied to spin-echo MRIs acquired using 1.5 T or 3 T MRI scanners from different manufacturers (slice thickness and resolution not reported).

ROCCELLA was a 52-week, randomized, double-blind phase 2 study that evaluated the efficacy and safety of s201086/GLPG1972 as a potential DMOAD in 932 knee OA patients [38, 95]. By enrolling patients with KLG 2 or 3 in combination with the presence of medial JSN 1 or 2, the study successfully enrolled knees with a sufficient loss in cartilage thickness to enable assessment of a structural benefit of s201086/GLPG1972 vs. placebo over 52 weeks. Still, ROCCELLA showed no effect on change from baseline to week 52 in the central medial femorotibial cartilage thickness (primary endpoint, based on manual, quality-controlled cartilage segmentations from sagittal gradient echo scans acquired using 1.5 T or 3 T scanners from different manufacturers, slice thickness: 1.5 mm, in-plane resolution: ~ 0.3 mm) or other outcomes (including pain measures and radiographic JSW). The selection criteria used for ROCCELLA may, however, be helpful for future clinical trials.

Several clinical trials evaluated the safety and efficacy of the injection of platelet-rich plasma (PRP) as a potential OA treatment, but only one of these trials assessed the potential structural efficacy using cartilage morphometry. The RESTORE trial evaluated the effects of intra-articular PRP injections in patients with symptomatic mild to moderate radiographic medial knee OA in 288 patients over 12 months [56]. When compared to placebo, the RESTORE trial found no significant differences in symptoms or change in medial tibial cartilage volume, which was measured manually from gradient echo MRI acquired on 3 T Philips or Siemens scanners (slice thickness: 0.5–0.6 mm, in-plane resolution: 0.5 × 0.5 to 0.66 × 0.63 mm).

Although these recent clinical trials employing quantitative cartilage morphology measurements as outcome were well designed and conducted, only two of the trials were able to demonstrate structural benefits based on different MRI protocols and different image analysis techniques and only one of these trials was able to demonstrate a dose–response effect on structural progression until today.

Based on the observation that cartilage thickness loss is only weakly associated with worsening of knee pain, a recent study raised the question, whether pain reduction can actually be achieved by chondroprotection [100]. This weak association may, however, be due to the relatively low baseline WOMAC pain severity in this cohort (average: 2.4 ± 3.1, scale 0…20), which also increased only slightly over 2 years (0.62 ± 3.4). In contrast to this study, the results from the Sprifermin subgroup at risk of progression demonstrated that pain reduction and structural benefits can be achieved by successfully stopping or even reverting cartilage loss in patients with severe baseline pain (median: 54, scale 0…100) [98]. Future studies aiming for the translation of a structural treatment effect into a clinical benefit should therefore most likely focus on knees with severe pain.

## Future directions

MRI and cartilage morphometry are still not accepted as primary outcome measures by regulatory agencies (FDA and EMA) for approval of cartilage-targeting molecules. Therefore, the qualification of MRI-based, direct measures of change in cartilage morphology as outcome measure for clinical trials is of great importance. This would allow to overcome the limitations of using radiographic JSW loss (e.g., impact of meniscus integrity and extrusion [10, 11] or the impact of knee positioning and x-ray beam alignment) as an indirect measure of cartilage loss.

Machine-learning techniques are increasingly used in OA research [46, 101] and have been used to identify predictors for structural OA progression [46, 101–103]. However, most of these studies relied on radiographic inclusion criteria of patients and radiographic instead of MRI-based measures of progression as outcome. More sophisticated machine learning models are likely to emerge, that may be able to provide reliable predictions of structural progression. Such prediction models will be valuable for enrolling patients likely to show symptom worsening and sufficient cartilage loss over the course of a trial. Future studies should also aim for directly predicting MRI-based cartilage loss instead of worsening of radiographic outcomes. After successful development and approval of a therapy with disease-modifying properties, automated segmentation techniques based on machine-learning may eventually allow the application of quantitative cartilage morphometry for treatment monitoring in individual OA patients. Cartilage morphometry is currently mostly used in knees with definite radiographic OA, with the presence of definite radiographic OA being the most widely used selection criterion for compiling cohorts likely to show structural progression [14, 79, 94]. More recently, prevalent MRI-based baseline cartilage damage has been shown to be predictive of subsequent further cartilage loss [104, 105] and has also been reported to occur in knees without definite radiographic OA [106]. More sophisticated selection criteria, such as simplified and less time-consuming MRI-based assessment of joint pathologies, machine-learning-based models, or dedicated early OA models such as the presence of radiographic OA in the contralateral knee [107], may allow to eventually also apply cartilage morphometry in the context of early OA or preventive studies.

## Summary

MRI-based cartilage morphometry has been demonstrated to be sensitive to OA-related change and also to treatment interventions and is nowadays widely used as an outcome measure in observational studies as well as interventional trials. Suitable imaging sequences are available for most of the 1.5 T and 3 T scanners currently used in clinical routine and have been validated to provide a high accuracy and reproducibility. Currently established predictors allow the selection of knees likely to show subsequent progression over the course of a clinical trial. Still, only few of the DMOAD trials were able to demonstrate structure modification of the drug candidates and no DMOAD has been approved by regulatory authorities yet. Lessons learned from previous trials and more sophisticated participant selection criteria (potentially based on machine-learning models) are likely to provide valuable guidance for future clinical trials relying on MRI-based cartilage morphometry as structural outcome measure.
